# Knowledge and attitude of human Mpox viral infection among pharmacy students in Jazan University: a web based cross-sectional study

**DOI:** 10.3389/fpubh.2025.1521923

**Published:** 2025-04-25

**Authors:** Eman Merghani Ali, Amani Khardali, Nawazish Alam, Abdulkarim M. Meraya, Hilal A. Thaibah, Dalin A. Hassan, Mohamed Eltaib Elmobark, Alanood Aladwani, Anugeetha Thacheril Mohanan, Hanan A. Bakri, Zamzam O. Mashraqi, Areej Mousa Salhabi

**Affiliations:** ^1^Department of Clinical Practice, Faculty of Pharmacy, Jazan University, Jazan, Saudi Arabia; ^2^Pharmacy Practice Research Unit (PPRU), Department of Clinical Practice, Faculty of Pharmacy, Jazan University, Jazan, Saudi Arabia; ^3^Department of Pharmacology and Toxicology, Faculty of Pharmacy, Jazan University, Jazan, Saudi Arabia; ^4^Department of Pharmaceutics, Faculty of Pharmacy, Jazan University, Jazan, Saudi Arabia; ^5^Pharmaceutical Care Department, King Fahd Central Hospital, Jazan, Saudi Arabia; ^6^Pharmacy Department, Wadi Al-Dawaser Armed Forced Hospital, Wadi Al-Dawasir, Saudi Arabia

**Keywords:** knowledge, attitude, students, monkeypox, Jazan

## Abstract

**Introduction:**

Saudi Arabia has witnessed the first confirmed case of Mpox on July 14th, 2022. Currently, there is no approved medication for the treatment of this infection. Therefore, prevention of this infection is crucial. This study aimed to assess knowledge and attitude toward the monkeypox viral infection among pharmacy students of Jazan University in Saudi Arabia.

**Methods:**

This is a cross-sectional, self-administered web-based study between the periods from April 2024 to June 2024. Descriptive statistics for all variables used chi-square statistics, and multivariate analysis to establish the association between participant’s demographic characteristics and knowledge of monkeypox disease.

**Results:**

The overall level of knowledge and attitude found 32 and 44.5%, respectively among pharmacy students. The source of information was commonly social media (55.5%), and only 38% reported receiving information during medical education. The factors associated with knowledge level included gender (*p* = 0.02), obesity (*p* = 0.03), receiving information from family or friends (*p* = 0.03), and during medical education (*p* = 0.016). The factors that were associated with attitude included age (*p* = 0.03), chronic disease (*p* = 0.0001), social media (*p* = 0.007), and medical education (*p* = 0.004) as sources of information.

**Conclusion:**

This study found the participants had a low level of knowledge and attitude toward Mpox infection. There is a need for the implementation of educational programs to know about this kind of outbreak and increase the knowledge and attitude of the students.

## Introduction

Human monkeypox (Mpox) is a zoonotic disease caused by Mpox virus (1). The Mpox virus belongs to the Poxviridae family and subfamily chordopoxvirinae, genus orthopoxivirus, and Monkeypox virus species (2). It is an encapsulated double-stranded DNA virus (3, 4). Sporadic outbreaks of the virus were reported in Africa due to contact with wild animals (5). Such outbreaks and the occurrence of travel-associated cases resulted in limited secondary transmission between humans (6). Mpox was known as a rare viral zoonosis that is endemic to Western and Central Africa however, it has evolved to become a global issue (7, 8). The World Health Organization (WHO) declared Mpox a public health emergency of international concern on July 23, 2022, to address the outbreak with the required urgency (9). The first confirmed case of human Mpox in Saudi Arabia was on July 14th, 2022, and by August 2022, there were five confirmed cases (10). In the past Jazan province of Saudi Arabia experienced a Rift Valley fever outbreak in 2000 with 500 cases and many deaths hence health science students must have appropriate knowledge about such kind of outbreak through health education or training (11).

The incubation period of the virus varies from five to 21 days, while signs and symptoms can persist between 2 to 5 weeks. The symptoms start with a fever, and other symptoms include back pain, muscle aching or myalgia, chills, headache, asthenia, swelling of lymph nodes, fatigue, and rashes (12, 13). Various-sized rashes appear within 1–5 days following fever, and they appear initially on the face and then include the rest of the body, legs, arms, and feet (14). The death cases ranged from 1 to 10 percent in epidemics, and the majority of cases occurred in children and young individuals (15). Currently, there is no drug has been approved by the US Food and Drug Administration (FDA) for the treatment of Mpox; even the antiviral tecovirimat, which has been approved for the treatment of smallpox such as, has no available data on its effectiveness in the treatment of Mpox (16).

Therefore, early detection and management, as well as the prevention of such infections, are necessary, and they represent a challenge for healthcare practitioners (17). The lack of knowledge is another major challenge in tackling this outbreak. Therefore, the assessment of the knowledge and attitude of the pharmacy students is necessary as they are the future healthcare professionals who are responsible for the management of the disease hence the lack of knowledge can be of adverse significance (18). Also, pharmacy students may affect the general population’s perception of the range of different diseases and improve their awareness regarding preventive measures (19). Therefore, this study was conducted to assess the knowledge and attitude of pharmacy students regarding monkeypox infection.

## Subjects and methods

### Design and procedure

This study is an analytical observational cross-sectional web-based survey study conducted on the pharmacy students of Jazan University, Saudi Arabia. The study was performed between the periods from April 2024 to June 2024. The study was conducted on pharmacy students who provided informed consent and at age 18 years and older. The study was conducted using a self-administered questionnaire to assess the knowledge and attitude of the students toward the monkeypox virus. The survey included questions that investigated the demographics of the students, their knowledge, and their attitude toward the monkeypox virus. The overarching goal of the study is to know the awareness among students of healthcare field with interdisciplinary understanding of public health issues.

### Study tools and data collection

Sample size was calculated by Raosoft sample size calculator. Confidence interval of 95% with a margin of 5% error. Approximately 400 undergraduate pharmacy studnets enrolled in first year to final year and a response distribution of 50%, a sample size of 188 was estimated. Data collection was carried out by a 43 item having 4 sections self-administered survey. Online surveys fulfilled the CHERRIES criteria. Multiple submission of participant’s response restricted and tracked with registered university email. Internal consistency was evaluated using Cronbach’s alpha, which yielded a value of 0.85. The questionnaire was validated by face as well as content validation. Bloom’s cut off point was determined to be used in this study with a score below 50% as low and score above 50% considered as high (20). Only completed questionnaires from participants were included in this study.

### Data analysis

SPSS software was used for data processing; continuous and qualitative variables were represented using mean (±SD) and number (%), respectively. Association between levels of knowledge and attitude with other variables were done, and *p* ≤ 0.05 was considered significant.

### Ethical consideration

The Research Ethics Committee of Jazan University approved this study. The approval number is REC 45/09/10–19, dated 24/03/2024. Participation in the study was entirely voluntary and free of coercion. Before starting the survey, all the participants provided their informed consent and confidentiality of participants were maintained.

## Results

A total of 200 students were included in this study; their characteristics are displayed in Table 1. The large majority of the students aged 18–25 years, 176 (88%), were females 167 (83.5%) and singles 160 (80%). More than one-half were from rural regions 107 (53.5%) and were in the fourth year of education 102 (51%). The largest proportion, 62 (31%), reported a family monthly income of 4–10.9 KSR. There, 35 (17.5%) reported having chronic disease, and 34 (17%) reported obesity. Regarding smoking, 25 (12.5%) were smokers.

**Table 1:** Socio-demographic/sample characteristics (*n* = 200).

Variables	Frequency (%)
Age in years	
18–25	176 (88)
26–35	17 (8.5)
> 36	7 (3.5)
Gender	
Male	33 (16.5)
Female	167 (83.5)
Marital status	
Single	160 (80)
Married	40 (20)
Residency	
Rural	107 (53.5)
Urban	93 (46.5)
Educational level	
1st year	36 (18)
2nd year	19 (9.5)
3rd year	22 (11)
4th year	102 (51)
5th year	21 (10.5)
Family monthly income	
< 4 K SR	50 (25)
4–10.9 K SR	62 (31)
11–15.9 K SR	45 (22.5)
>16 K SR	43 (21.5)
Chronic disease	
Yes	35 (17.5)
No	165 (82.5)
Obesity	
Yes	34 (17)
No	166 (83)
Smoking	
Yes	25 (12.5)
No	175 (87.5)

Five questions investigated information related to the virus (Table 2). The major source of information for the students was social media 111 (55.5%), followed by healthcare providers 64 (32%), whereas 5 (2.5%) of the students reported having no information. More than one-half, 132 (66%), reported being infected with COVID-19. The large majorities of the students reported administrating the COVID-19 vaccines 188 (94%) and completing the childhood vaccines 179 (89%). Only 76 (38%) reported receiving information about the human monkeypox virus during medical education.

**Table 2:** Participants information and awareness regarding viral outbreak.

Variables	Frequency (%)
Source of information (more than answer allowed)	
TV	40 (20)
Social media	111 (55.5)
Healthcare provider	64 (32)
Family or friends	46 (23)
Books	22 (11)
I have no information	5 (2.5)
Did you infect with COVID-19?	
Yes	132 (66)
No	68 (34)
Do you take two or more COVID-19 vaccines?	
Yes	188 (94)
No	12 (6)
Have you completed the childhood vaccines?	
Yes	178 (89)
No	22 (11)
Have you ever received information about the human monkeypox virus during medical education?	
Yes	76 (38)
No	124 (62)

**Figure 1:**
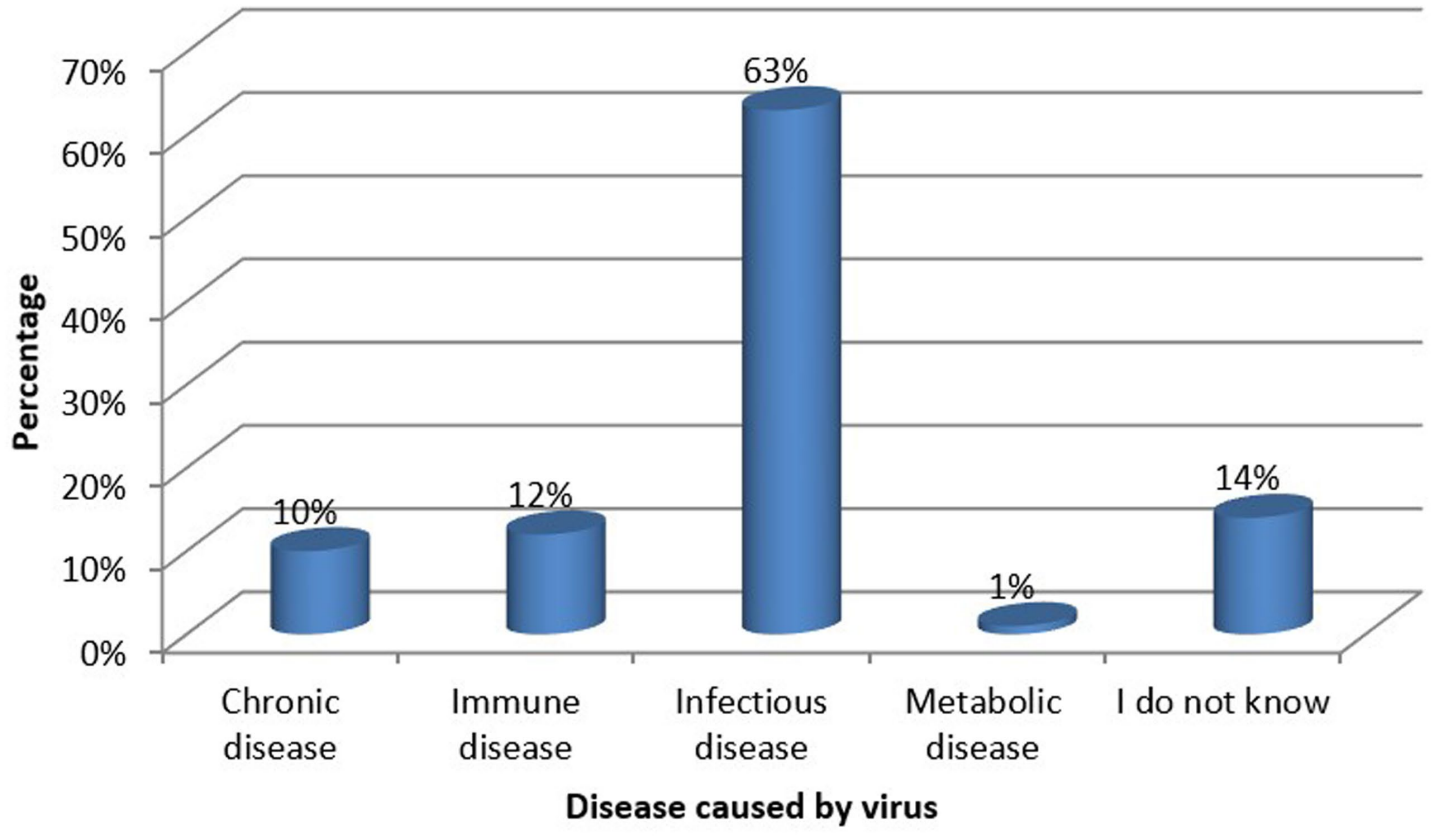
Disease caused by the virus according to knowledge of the students.

It was reported that the virus causes infectious diseases by 126 (63%) pharmacy students, whereas 28 (14%) did not know (Figure 1).

The students’ knowledge was investigated through 23 questions with three options as answers: either yes, no, or I do not know. The questions of knowledge and the answers of the students are shown in Table 3. The overall level of knowledge was high among 64 (32%), and it was low among more than one-half of the students 136 (68%).

**Table 3:** Descriptive of participant’s knowledge regarding human Mpox.

Questions	Yes n (%)	No n (%)	I do not know n (%)
Monkeypox virus is a new infection that outbreak in 2022	94 (47)	47 (23.5)	59 (29.5)
Chicken pox and monkeypox virus are the same disease	22 (11)	103 (51.5)	75 (37.5)
Monkeypox virus is common in Middle Eastern countries	43 (21.5)	67 (33.5)	90 (45)
Monkeypox virus is common in West and Central African	79 (39.5)	26 (13)	95 (47.5)
There are many cases recorded in Saudi Arabia	42 (21)	89 (44.5)	69 (34.5)
Monkeypox virus is a contagious viral disease	116 (58)	15 (7.5)	69 (34.5)
Monkeypox virus is a contagious bacterial disease	32 (16)	104 (52)	64 (32)
Monkeypox virus is transmitted to humans through bites and scratches from infected animals	86 (43)	32 (16)	82 (41)
The monkeypox virus is a sexually transmitted disease	56 (28)	62 (31)	82 (41)
The disease can be transmitted between humans	130 (65)	12 (6)	58 (29)
Monkeypox virus is spread by droplets (coughing and sneezing)	82 (41)	35 (17.5)	83 (41.5)
Monkeypox virus vertically from mother to child	47 (23.5)	38 (19)	115 (57.5)
Monkeypox virus can be transmitted via blood-borne transmission	46 (23)	42 (21)	112 (56)
The monkeypox virus can be transmitted through body fluid	80 (40)	19 (9.5)	101 (50.5)
Skin rash is a symptom of the monkeypox virus	119 (59.5)	18 (9)	63 (31.5)
The first symptoms of the monkeypox virus are similar to flu	84 (42)	31 (15.5)	85 (42.5)
The typical incubation period of monkeypox virus (5-21 days)	61 (30.5)	17 (8.5)	122 (61)
Hand sanitizers and face masks are important in preventing the monkeypox virus	109 (54.5)	20 (10)	71 (35.5)
Monkeypox virus outbreak in 2022 was noted related to homosexuality	75 (37.5)	29 (14.5)	96 (48)
MBV is detected by using Immunoglobulin (IgG/IgM) Antibodies in the blood samples	66 (33)	20 (10)	114 (57)
There is a monkeypox vaccine in Saudi Arabia	42 (21)	49 (24.5)	109 (54.5)
The chickenpox vaccine I got in childhood protects me from the monkeypox virus	39 (19.5)	48 (24)	113 (56.5)
There is a smallpox vaccine that can be used for monkeypox virus	52 (26)	31 (15.5)	117 (58.5)

The attitude of the students was investigated through seven questions; each question was provided with five answers: either strongly disagree, disagree, neutral, agree, or strongly agree. The questions about attitude and the students’ answers are shown in Table 4. The overall attitude of students was high among only 89 (44.5%), whereas 111 (56.5%) reported a low level of attitude.

**Table 4:** Descriptive of participant’s attitude regarding human Mpox.

Questions	Strongly disagree	Disagree	Neutral	Agree	Strongly agree
I believe that MPV prevention and control measures are adequately available.	43 (21.5)	66 (33)	69 (34.5)	13 (6.5)	9 (4.5)
I have negative feelings about MPV.	37 (18.5)	47 (23.5)	87 (43.5)	19 (9.5)	10 (5)
I think that MPV can be transmitted to KSA.	39 (19.5)	81 (40.5)	61 (30.5)	11 (5.5)	8 (4)
I believe that media coverage of MPV may have an impact on its global prevention	50 (25)	65 (32.5)	66 (33)	10 (5)	9 (4.5)
I think MPV will become a new pandemic, and its impact will be like COVID–19.	29 (14.5)	39 (19.5)	79 (39.5)	50 (25)	3 (1.5)
I believe that traveling to MPV-infected countries is risky.	62 (31)	63 (31.5)	65 (32.5)	3 (1.5)	7 (3.5)
I believe that travel – medicine should be a required course during my medical education.	58 (29)	70 (35)	56 (28)	5 (2.5)	11 (5.5)

The association between demographics and level of knowledge is shown in Table 5. There were significant association found between knowledge levels and each of gender (*p* = 0.02), marital status (*p* = 0.01), family income (*p* = 0.009), and obesity (*p* = 0.03).

**Table 5:** Association between socio-demographics and knowledge level.

	Knowledge level		
	High (>50%; *n* = 64)	Low (<50%; *n* = 136)	*p* value
Age in years			
18–25	60 (93.8)	116 (85.3)	0.122
26–35	4 (6.3)	13 (9.6)	
> 36	0 (0)	7 (5.1)	
Gender			
Male	16 (25)	17 (12.5)	0.026*
Female	48 (75)	119 (87.5)	
Marital status			
Single	58 (90.6)	102 (75)	0.010*
Married	6 (9.4)	34 (25)	
Residency			
Rural	31 (48.4)	76 (55.9)	0.325
Urban	33 (51.6)	60 (44.1)	
Educational level			
1st year	16 (25)	20 (14.7)	0.359
2nd year	5 (7.8)	14 (10.3)	
3rd year	5 (7.8)	17 (12.5)	
4th year	33 (51.6)	69 (50.7)	
5th year	5 (7.8)	16 (11.8)	
Family monthly income			
< 4 K SR	22 (34.4)	28 (20.6)	0.009*
4–10.9 K SR	10 (15.6)	52 (38.2)	
11–15.9 K SR	15 (23.4)	30 (22.1)	
>16 K SR	17 (26.6)	26 (19.1)	
Chronic disease	13 (20.3)	22 (16.2)	0.473
Obesity	16 (25)	18 (13.2)	0.039*
Smoking	6 (9.4)	19 (14)	0.359

*p ≤ 0.05.

The association between the knowledge of the students and other factors are shown in Table 6. Significant association were found between knowledge levels and various factors, including family or friends as the source of information (*p* = 0.039), infection with COVID-19 (*p* = 0.002), receiving COVID-19 vaccine (*p* = 0.01), and receiving information about human monkeypox virus (*p* = 0.016).

**Table 6:** Association between awareness and knowledge level.

Variables	Knowledge level		*p* value
	High (>50%; *n* = 64)	Low (<50%; *n* = 136)	
Source of information			
TV	12 (18.8)	28 (20.6)	0.762
Social media	34 (53.1)	77 (56.6)	0.643
Healthcare provider	23 (35.9)	41 (30.1)	0.413
Family or friends	9 (14.1)	37 (27.2)	0.039*
Books	5 (7.8)	17 (12.5)	0.323
I have no information	3 (4.7)	2 (1.5)	0.330
Did you infect with COVID-19	52 (81.3)	80 (58.8)	0.002*
Do you take two or more COVID–19 vaccines	64 (100)	124 (91.2)	0.010*
Do you complete the childhood vaccines	57 (89.1)	121 (89)	0.985
Have you ever received information about the human monkeypox virus during medical education	32 (50)	44 (32.4)	0.016*

*p ≤ 0.05.

The association between levels of attitude and demographics of the students are shown in Table 7. Significant associations with attitude were found regarding age (*p* = 0.03), family income (*p* = 0.0001), and having chronic disease (*p* = 0.0001).

**Table 7:** Association between socio-demographics and attitude level.

Variables	Attitude level		*p* value
	High (>50%; *n* = 89)	Low (<50%; *n* = 111)	
Age in years			
18–25	79 (88.8)	97 (87.4)	0.030*
26–35	10 (11.2)	7 (6.3)	
>36	0 (0)	7 (6.3)	
Gender			
Male	12 (13.5)	21 (18.9)	0.303
Female	77 (86.5)	90 (81.1)	
Marital status			
Single	75 (84.3)	85 (76.6)	0.176
Married	14 (15.7)	26 (23.4)	
Residency			
Rural	42 (47.2)	65 (58.6)	0.109
Urban	47 (52.8)	46 (41.4)	
Educational level			
1st year	17 (19.1)	19 (17.1)	0.176
2nd year	8 (9)	11 (9.9)	
3rd year	15 (16.9)	7 (6.3)	
4th year	40 (44.9)	62 (55.9)	
5th year	9 (10.1)	12 (10.8)	
Family monthly income			
< 4 K SR	14 (15.7)	36 (32.4)	0.000*
4–10.9 K SR	37 (41.6)	25 (22.5)	
11–15.9 K SR	14 (15.7)	31 (27.9)	
>16 K SR	24 (27)	19 (17.1)	
Chronic disease	6 (6.7)	29 (26.1)	0.000*
Obesity	16 (18)	18 (16.2)	0.742
Smoking	8 (9)	17 (15.3)	0.179

*p ≤ 0.05.

Also, other factors displayed significant correlations with attitude levels, including social media as a source of information (*p* = 0.007), receiving the COVID-19 vaccine (*p* = 0.0001), and receiving information about human monkeypox virus (*p* = 0.004; Table 8).

**Table 8:** Association between awareness and attitude level.

Variables	Attitude level		*p* value
	High (>50%; *n* = 89)	Low (<50%; *n* = 111)	
Source of information			
TV	18 (20.2)	22 (19.8)	0.943
Social media	40 (44.9)	71 (64)	0.007*
Healthcare provider	31 (34.8)	33 (29.7)	0.442
Family or friends	22 (24.7)	24 (21.6)	0.605
Books	10 (11.2)	12 (10.8)	0.924
I have no information	2 (2.2)	3 (2.7)	1.000
Did you infect with COVID-19	55 (61.8)	77 (69.4)	0.261
Do you take two or more COVID–19 vaccines	78 (87.6)	110 (99.1)	0.000*
Have you completed the childhood vaccines	79 (88.8)	99 (89.2)	0.924
Have you ever received information about the human monkeypox virus during medical education:	24 (27)	52 (46.8)	0.004*

*p ≤ 0.05.

## Discussion

This study aimed to assess the knowledge and attitude of pharmacy students regarding monkeypox infection. There is a lack of Saudi studies conducted on this subject that are focused on pharmacy students. In the current study, the overall knowledge of the students was low as higher proportions of students had a low overall level of knowledge.

A previous Saudi study conducted on healthcare practitioners and students revealed that the majority of the participants provided correct answers about the type of microorganisms that cause human Mpox infection. Also, they displayed good knowledge regarding the common symptoms but had poor knowledge regarding the less common symptoms. Additionally, 70% knew the effectiveness of antiviral drugs to treat Mpox (21). The study reported that the participants had gaps in knowledge, with a median score of knowledge of 5 out of 6 as the maximum score. In our study, more than one-half reported that the disease is a contagious viral disease, and 52% did not agree that it is a bacterial disease. Also, more than one-half of the students (59.5%) stated that skin rash is a symptom of the Mpox virus, and 42% reported that the first symptoms are similar to flu. Therefore, the findings in our study reflect gaps in the knowledge of students similar to the previous Saudi study. Additionally, the previous Saudi study found that the knowledge of the participants was independently associated with the age of the participants, where the students and health practitioners aged 30–49 years and 50–69 years reported higher scores of knowledge (22). The contrast was found in our study as there was no significant difference in the level of knowledge between different age groups of the students (*p* = 0.1).

Similar to our study, a previous United Arab Emirates (UAE) study conducted on medical students reported an inadequate level of knowledge regarding Mpox infection among humans. The mean score of knowledge was 73.95, which indicated a moderate level of knowledge and indicated that 49.3% of the students had good knowledge. There was a significant association between the knowledge of students and receiving information on this viral infection during their education (*p*<0.01) and seniority (*p*<0.01) (23). Therefore, it seems that the lack of knowledge among medical students is not restricted to Saudi Arabia alone but to other Arabian countries. In agreement with the previous study there was a significant association between the level of knowledge and receiving information about human Mpox during medical education (18), but in contrast to the previous study, we did not find any association between the level of knowledge and the education level.

In Pakistan, a study enrolled medical, pharmacy, and nursing students, which demonstrated that 21.6% had good knowledge, 43.2% had moderate knowledge, and 35.2% had poor knowledge. Additionally, the factors that significantly affected the level of knowledge were age (*p* = 0.01), education (*p*<0.001), and gender (*p*<0.001). However, in multivariate analysis, education was the only predictor of knowledge (21). The current study showed a lower level of as only 32% had a good level of knowledge, whereas more than half of the students (68%) had poor knowledge. Additionally, we did not find any association between the knowledge level and age or education level; however, in agreement with the previous study, we found that gender was associated with the level of knowledge, and females significantly tended to have lower levels of knowledge.

Another study from Pakistan conducted on University students and reported knowledge and attitude found that the overall knowledge of Mpox was average, with the presence of knowledge gaps in most aspects, whereas the overall attitude was neutral (22). Our study displayed worse findings, where the highest proportions of the students had low knowledge and low attitude. Additionally, we did not find any association between knowledge and residency of the students (*p* = 0.3), whereas the previous study reported a strong association between the knowledge of the students and region of respondents (24). Additionally, the age of the students was significantly associated with their attitude, and this was in agreement with our findings, where a significant association was found between knowledge and age in our study.

The variations in the levels of knowledge and attitude between our study and other studies from other countries and even the studies conducted on students from other Saudi Regions aren’t surprising. This can be explained by the fact that there are variations in the level of education and information the students receive in each country. Additionally, a previous study conducted on 11,919 medical students from 27 countries revealed that only 12.5% had training on Mpox, and only 55.3 and 51.7% had good knowledge and positive attitude toward Mpox (19). Therefore, our findings seem to be similar to the global findings regarding the low level of knowledge and attitude regarding Mpox among pharmacy students. Additionally, the positive predictors of good knowledge included obtaining information from friends, research articles, scientific websites, and social media. On the other hand, male gender had a negative association with good knowledge of Mpox (19). Despite the fact that we did not perform multivariate analysis, we found that gender had no significant association with knowledge. However, obtaining information from family and friends was associated with a low level of knowledge, which was in contrast to the previous study. Regarding attitude, being in urban regions and in the fifth year of medical education, receiving information from social media or scientific websites was associated with a positive attitude, whereas being male was negatively associated with a positive attitude. Our findings were different as residency region, educational level, and gender had no significant association with attitude. Furthermore, receiving information from social media was significantly associated with a low level of attitude.

There is a lack of studies assessing the attitude of medical/pharmacy students regarding Mpox. A previous Saudi study assessed the knowledge and attitude of healthcare workers regarding Mpox, and it was found that the majority of respondents had inadequate knowledge, but they had positive attitudes (25). In our study, both knowledge and attitude were low among the students. A study from Bangladesh conducted on nurses reported inadequate knowledge but high and positive attitude among the nurses, where the female gender was associated with better knowledge and more positive attitude (26). In our study, the female gender was associated with a low level of knowledge, and both genders had no significant variation regarding the level of attitude. There is a need for development of unified zoonotic disease surveillance system under the one health spectrum to effectively control the spread of Mpox and one health approach need to be adopted to address the crisis for special population and minimize the such outbreak in future (27, 28). The implication of this study suggest that targeted training and educational programs should be developed and implemented. Importantly, our study also highlights the need for increased education and awareness about monkeypox disease among pharmacy students in the Jazan University. By taking these steps, we can better prepare for future outbreaks and ensure the health and safety of the community.

### Limitations of the study

One of the potential limitation include this study is being conducted among Jazan University pharmacy students only. Hence we cannot generalize the results to other university pharmacy student’s or other parts of country’s pharmacy students. Another limitation is the study sample could not be considered representative, due to convenience sampling and the potential for selection bias cannot be ruled out due to convenience sampling.

## Conclusion

In this study, we investigated the knowledge and attitude of pharmacy students in the Jazan University, Saudi Arabia, toward monkeypox disease and its prevention. The pharmacy students in this study had a low level of knowledge and attitude toward Mpox. The lack of knowledge and negative attitude regarding Mpox seems to be a global issue among pharmacy students and even healthcare workers. Therefore, there is a great need for the establishment of educational programs incorporating pharmacy students and healthcare workers to increase their knowledge and attitude toward Mpox in order to prevent Mpox infections and appropriately manage the infection as possible. Additionally, the factors affecting the knowledge and attitude of the pharmacy students varied, and investigations of further factors are required. In contrast to the health education and health promotion comprehensive One health approach, awareness raising program need to be adopted.

### Future scope

Since this study includes only Jazan University pharmacy students hence we cannot generalize the findings. Future research possibilities are to include other university pharmacy students on large sample size. Moreover, qualitative research should be administered to obtain reasonable, broad and more thorough understanding of the knowledge and attitude of human monkeypox viral infection among pharmacy students in Saudi Arabia.

## Data Availability

The raw data supporting the conclusions of this article will be made available by the authors, without undue reservation.
